# Regional Variation in Supply and Use of Psychiatric Services in 3 Canadian Provinces: Variation régionale de l’offre de services psychiatriques et de leur utilisation dans trois provinces canadiennes

**DOI:** 10.1177/07067437251322404

**Published:** 2025-03-28

**Authors:** David Rudoler, Ridhwana Kaoser, M Ruth Lavergne, Sandra Peterson, James M. Bolton, Matt Dahl, François Gallant, Kimberley P Good, Myriam Juda, Alan Katz, Jason Morrison, Benoit H. Mulsant, Alison L Park, Philip G Tibbo, Juveria Zaheer, Paul Kurdyak

**Affiliations:** 1 Faculty of Health Sciences, Ontario Tech University, Oshawa, Ontario, Canada; 2 25487 Ontario Shores Centre for Mental Health Sciences, Whitby, Ontario, Canada; 3 Faculty of Health Sciences, 1763 Simon Fraser University at Harbour Centre, Vancouver, British Columbia, Canada; 4 Department of Family Medicine, 12361 Dalhousie University, Halifax, Nova Scotia, Canada; 5 Centre for Health Services and Policy Research, School of Population and Public Health, 8166 The University of British Columbia, Vancouver, British Columbia, Canada; 6 Department of Psychiatry, 8664 University of Manitoba, Winnipeg, Manitoba, Canada; 7 Unité de Médecine Familiale, Dieppe, New Brunswick, Canada; 8 Department of Psychiatry, 3688 Dalhousie University, Halifax, Nova Scotia, Canada; 9 Department of Psychology and Neuroscience, 3688 Dalhousie University, Halifax, Nova Scotia, Canada; 10 Telfer School of Management, 6363 University of Ottawa, Ottawa, Ontario, Canada; 11 Department of Community Health Sciences, Rady Faculty of Health Sciences, 8664 University of Manitoba, Winnipeg, Manitoba, Canada; 12 Department of Family Medicine, Rady Faculty of Health Sciences, 8664 University of Manitoba, Winnipeg, Manitoba, Canada; 13 Nova Scotia Health, Halifax 3688 , Nova Scotia, Canada; 14 Department of Psychiatry, University of Toronto, Toronto, Ontario, Canada; 15 ICES, Toronto, Ontario, Canada; 16 Nova Scotia Health Authority, Halifax, Nova Scotia, Canada; 17 Centre for Addiction and Mental Health, Toronto, Ontario, Canada

**Keywords:** mental health services, psychiatry, workforce, health services accessibility, physician practice patterns, physician supply and distribution

## Abstract

**Objective:**

To examine the patterns in the supply and use of psychiatric services in 3 Canadian provinces: British Columbia, Manitoba, and Ontario.

**Methods:**

We conducted a repeated cross-sectional analysis spanning fiscal years 2012/13 to 2021/22, using patient- and psychiatrist-level data aggregated into administrative health regions. Descriptive statistics and linear regression were used to assess patterns and relationships between the per capita number of psychiatrists (“supply”) and measures of use of psychiatric services (“utilization”), including any psychiatrist contact, psychiatric consultation (1–2 visits with the same psychiatrist), and ongoing psychiatric care (3 or more visits with the same psychiatrist).

**Results:**

The number of psychiatrists per capita remained stable within the 3 provinces during the study period. In 2021/22, Vancouver had the highest number in British Columbia (45 psychiatrists per 100,000 individuals), compared to 14 per 100,000 in lower-supply regions. Toronto had the highest number in Ontario (38 per 100,000), compared to 9 in lower supply regions. Winnipeg had the highest number in Manitoba (25 per 100,000), compared to 7 in the lower supply regions. In 2021/22, the per capita number of psychiatrists was moderately correlated with any psychiatrist contact (*R*^2^ = 0.290) and ongoing psychiatric care (*R*^2^ = 0.411), but weakly correlated with psychiatric consultation (*R*^2^ = 0.005). The relationship between supply and utilization diminishes with higher levels of regional supply.

**Conclusions:**

Psychiatrists were unevenly distributed within and across provinces. While more psychiatrists are needed, the moderate and diminishing relationships between their numbers and utilization suggest that increasing this number alone is unlikely to fully address unmet needs for mental healthcare. Strategies to improve access will need to directly target uneven distributions. Further research is needed to understand the factors influencing psychiatrists’ practice choices and ways to better support them in increasing their access to care.

## Résumé

**Objectif:**

examiner les tendances de l’offre de services psychiatriques et de leur utilisation dans trois provinces canadiennes : Colombie-Britannique, Manitoba et Ontario.

**Méthodologie:**

nous avons mené une analyse transversale répétée couvrant les exercices 2012-2013 à 2021-2022, en utilisant des données au niveau des patients et des psychiatres, agrégées en régions sanitaires. Des statistiques descriptives et des régressions linéaires ont été utilisées pour évaluer les tendances et les relations entre le nombre de psychiatres par habitant (« offre ») et les mesures de l’utilisation des services psychiatriques (« utilisation »), y compris les contacts avec un psychiatre, les consultations psychiatriques (une à deux visites avec le même psychiatre) et les soins psychiatriques continus (trois visites ou plus avec le même psychiatre).

**Résultats:**

le nombre de psychiatres par habitant est demeuré stable dans les trois provinces au cours de la période étudiée. En 2021-2022, Vancouver comptait le nombre le plus élevé de psychiatres en Colombie-Britannique (45 pour 100 000 personnes), comparativement à 14 pour 100 000 dans les régions où l’offre était plus faible. C’est à Toronto que l’on trouvait le nombre le plus élevé de psychiatres en Ontario (38 pour 100 000), contre neuf dans les régions où l’offre était plus faible. Winnipeg enregistrait le nombre le plus élevé de psychiatres au Manitoba (25 pour 100 000), comparativement à sept dans les régions où l’offre était plus faible. En 2021-2022, le nombre de psychiatres par habitant était modérément corrélé aux contacts avec un psychiatre (*R*^2^ = 0,290) et aux soins psychiatriques continus (*R*^2^ = 0,411), mais faiblement corrélé aux consultations psychiatriques (*R*^2^ = 0,005). La relation entre l’offre et l’utilisation diminue avec l’augmentation de l’offre régionale.

**Conclusions:**

les psychiatres étaient inégalement répartis à l’intérieur des provinces et entre elles. Bien qu’il faille davantage de psychiatres, les relations modérées et décroissantes entre leur nombre et l’utilisation des services laissent à penser que l’augmentation de ce nombre ne suffira probablement pas à répondre aux besoins non satisfaits en matière de soins de santé mentale. Les stratégies visant à améliorer l’accès devront cibler directement la répartition inégale. Des recherches supplémentaires sont nécessaires pour comprendre les facteurs qui influencent les choix de pratique des psychiatres et les moyens de les soutenir davantage pour qu’ils améliorent l’accès aux soins.

## Introduction

Physicians are an essential but partial element of a well-functioning mental health care system. However, provincial public health insurance systems in Canada typically cover only medically necessary hospital and physician services, whereas most other mental health services are paid privately. Those relying on public coverage often have limited options beyond publicly funded physician services for timely, specialized outpatient mental healthcare.^1^

The use of mental health services has been increasing over time.^2^ Between 2009 and 2017, outpatient mental health visits and psychiatric hospitalizations in Ontario increased by 9% and 23%, respectively. Meanwhile, emergency department visits for self-harm also increased by 24%, particularly in younger populations.^3^ In Manitoba, the overall prevalence of mental illnesses remained stable over time, and the prevalence of most mental illnesses was higher in urban areas compared to rural areas.^4^ Northern areas of the province reported higher suicide and attempted suicide rates.^4^ Mental health services also increased during and after the COVID-19 pandemic. For instance, between 2019 and 2021, mental health-related outpatient visits increased by 24% in British Columbia.^5^ These trends put pressure on the already stretched mental healthcare resources.

Access to outpatient mental health services is limited, particularly among those with high needs.^6,7^ For instance, in Ontario, about 70% of people do not see a psychiatrist in the 30 days following psychiatric hospitalization discharge, and about 60% do not see one within 6 months of a suicide attempt.^7^ In British Columbia, more than 50% of people who visit the emergency department multiple times for mental health services were not seen by an outpatient psychiatrist in the previous year.^8^ However, timely follow-up care reduces hospital readmission risk,^9,10^ and poor access to mental healthcare can increase the risk of relapse, readmission, avoidable healthcare costs, and mortality.^11,12^

Previous research from Ontario suggests that there is wide variation in the per capita supply of psychiatrists across provinces, with densely populated urban areas having the highest supply regions.^13^ Thus, increasing the per capita number (“supply”) of psychiatrists, particularly in rural regions, may address these unmet needs. However, studies have also suggested that high per capita supply may not necessarily translate into increased access. For instance, research conducted in Ontario suggests that a large proportion of psychiatrists practicing in high per capita supply regions tend to see fewer than 100 unique patients per year.^13^ A follow-up study showed that about a quarter of psychiatrists practicing in Ontario saw fewer than 2 new patients per month.^14^ These studies challenged the idea that increasing per capita supply alone would solve issues with unmet needs for community-based mental healthcare.

Understanding the patterns of psychiatric care use and supply is key to informing human resource planning and interventions that can improve access to care. However, much of the research and analysis in this area focuses on the province of Ontario.^7,13–16^ There is uncertainty regarding the supply and use of psychiatric care in other jurisdictions in Canada. National reporting on the supply of mental health and substance use healthcare providers shows variation in per capita supply,^17^ but there is limited data on the relationship between supply and use and intra-provincial variation in supply and use of psychiatric care. Several factors could contribute to regional variation, including differences in geography, demographics, and provincial policy interventions (e.g., fee schedules and incentives).

This study examined the number of psychiatrists and the patterns of psychiatric services in 3 Canadian provinces: British Columbia (BC), Manitoba (MB), and Ontario (ON). We developed and analyzed comparable measures of the use of psychiatric services from administrative health data in these provinces. We also assessed the relationship between the number of psychiatrists (“supply”) within regions and regional rates of use of psychiatric services (“utilization”). Understanding these patterns will provide information that could inform human resource planning in mental health care sectors.

## Methods

### Setting and Study Design

We conducted a repeated cross-sectional study from fiscal year (FY) 2012/13 to FY 2021/22. We analyzed psychiatric care utilization and provision in 3 Canadian provinces: BC, MB, and ON. We aggregated patient- and psychiatrist-level data into administrative health regions in these provinces. The 16 Health Service Delivery Areas (HSDA) in BC, 5 Regional Health Authorities (RHA) in Manitoba, and 35 public health units (PHU) in Ontario were the units of analysis. This study was approved by the Ontario Tech University Review Board (REB number: 16637), Simon Fraser University and University of British Columbia harmonized review boards (REB number: H21–03694), and the University of Manitoba Health Review Board (HREB number: HS25310 (H2022:010)).

### Data Sources

This study received in-kind coordination support from the Health Data Research Network (HDRN) and Canada's Data Access Support Hub (DASH) to facilitate administrative data access across regions. HDRN Canada is supported by the Canadian Institutes of Health Research (CIHR) under Canada's Strategy for Patient-Oriented Research (SPOR). We used health administrative databases from Population Data BC, Manitoba Centre for Health Policy (MCHP), and ICES (Ontario) to create consistent variable definitions (Table A1 in the Supplemental Material). Data from Ontario were linked using unique encoded identifiers and analyzed at ICES. The data covered all practising psychiatrists and patients’ interactions during the study period.

### Participants

We generated separate data files containing population and psychiatrist data for each fiscal year during the study period. The population files included adults (18–105 years of age) registered for 1 or more fiscal years with 1 of the provincial health insurance systems in 3 provinces. We excluded individuals who died before April 1 of each fiscal year from the corresponding file. The psychiatrist-level files included all physicians specializing in psychiatry who were registered with a provincial regulatory college or who had billed the health insurance system as psychiatrists during the study period. Physicians with no claims as psychiatrists during the fiscal year were excluded from the corresponding file. Subsequently, we aggregated population and psychiatrist data at the regional administrative health level. We assigned the patients to a region based on their location of residence. Psychiatrists were assigned to the region where the plurality of patients they saw lived during the fiscal year.

### Measures of Use of Psychiatric Services

We developed measures for the use of psychiatric services (“utilization”) in consultation with clinical experts. We prioritized measures that captured the core aspects of psychiatric practice and were consistently quantifiable across the provinces. Specifically, we measured *any psychiatrist contact* as the number of unique patients seen by a psychiatrist (in any location, including the office, emergency department, and hospital), divided by the adult population eligible for provincial health insurance within a region. *Psychiatric consultation* was defined as the number of unique patients with 1 or 2 outpatient visits by the same psychiatrist, divided by the eligible adult population within a region. *Ongoing psychiatric care* was defined as the number of unique patients with 3 or more outpatient visits annually with the same psychiatrist, divided by the eligible adult population within a region.

### Regional Characteristics

We characterized the regions in each province by the per capita number of psychiatrists (psychiatrists per 100,000). We also classified regions into different levels of rurality using Statistical Area Classification (SAC) as “Metro” (SAC type 1), “Other Urban” (SAC types 2 or 3), and “Rural” (SAC types 4 to 7) based on the predominant SAC types of regional residents. As crude proxies for the regional need for services from psychiatrists, we measured rates of use of mental health or substance use services in any service location, rates of hospitalization for mental disorders, hospitalization for substance use disorders, and the regional prevalence of schizophrenia and related disorders.^18^ See Table A2 in the Supplemental Material for more details.

### Statistical Analysis

We used simple linear regression to assess the relationships between regional characteristics and per capita number of psychiatrists, per capita supply and utilization, and between utilization and regional characteristics. Unless otherwise specified, we weighted all regressions by the regional population. We reported the *R*^2^ statistics to assess the strength of these relationships. Given that we observed the full population of billed psychiatric services, we did not report the inferential statistics. We performed all analyses for the general regional adult population (18–105 years of age) and a subpopulation of adults diagnosed with schizophrenia or related disorders.^18^

### Results

The per capita number of psychiatrists was stable within the 3 study provinces between FY 2012/13 and FY 2021/22 (Figure A1 in Supplementary Material). Trends in any psychiatrist contact, psychiatric consultation, and ongoing psychiatric care were also stable (see Figure A2 in the Supplementary Material). Thus, we report results only for FY 2012/13 and FY 2021/22. British Columbia had the highest supply per capita during this period, whereas Manitoba had the lowest. Table 1 shows the top 8 regions ranked according to per capita supply in FY 2021/22. Vancouver HSDA had the highest supply in British Columbia, with 45 psychiatrists per 100,000 population, compared with 14 per 100,000 population in the lower supply regions of the province. Toronto PHU had the highest supply in Ontario, with 38 per 100,000 population, compared to 9 per 100,000 population in lower-supply regions. The Winnipeg RHA had the highest supply in Manitoba, with 25 per 100,000 population, compared to 7 per 100,000 population in lower-supply regions.

**Table 1.** Regional Characteristics (FY 2021/22).

Variable	Vancouver HSDA (32)	Toronto PHU	Kingston PHU	Ottawa PHU	South Vancouver Island HSDA (41)	London PHU	Hamilton PHU	Winnipeg RHA (5)	Low supply BC^a^	Low supply ON^a^	Low supply MB^a^	*R*-squared^b^
Total adult population	638,384	2,598,967	182,980	890,663	372,589	424,795	498,895	655,553	3,454,186	8,022,351	452,680	–
Number of psychiatrists	290	990	60	279	108	117	128	161	470	610	32	–
Psychiatrists per 100,000	45.427	38.092	32.790	31.325	28.986	27.543	25.657	24.559	13.607	9.486	7.069	0.826^c^
% MHSU users	18.014	18.966	21.144	19.550	22.128	20.266	22.546	25.724	19.478	19.560	22.435	0.021
% lowest neighbourhood income quintile	24.395	33.033	22.869	17.349	20.663	20.709	25.769	20.014	20.295	15.463	21.220	0.392
% age 18–44	52.754	48.368	41.960	46.552	41.865	46.766	45.505	48.608	43.821	43.438	46.223	0.202
Substance use disorder service users per 1,000	17.294	11.393	18.095	10.207	16.826	15.356	19.766	8.895	15.050	14.031	8.905	<0.001
Schizophrenia diagnosis per 1,000	14.540	12.842	13.165	10.615	12.241	11.921	13.051	11.599	9.608	9.074	6.753	0.606
Mental health hospitalization per 10,000	36.467	28.042	34.813	28.136	34.381	39.737	29.024	31.714	36.544	31.107	26.796	<0.001
Substance use disorder hospitalization per 10,000	12.093	9.646	15.248	8.252	16.855	11.653	9.621	10.785	19.539	11.470	14.734	0.015
Rurality	Metro	Metro	Metro	Metro	Metro	Metro	Metro	Metro	–	–	–	–

^a^ Low-supply columns contain the average values for all regions outside of the top 8 regions in terms of per-capita supply. ^b^Unless otherwise noted, R-Squared values are for the relationship between per capita supply and the row header. ^c^Value represents the correlation between regional ranking of per-capita supply and per capita supply per 100,000.

We observed a positive relationship between per capita supply and the proportion of the population who lived in the lowest neighbourhood income quintile (*R*^2^ = 0.392) and the proportion between the ages of 18 and 44 (*R*^2^ = 0.202). We observed a positive relationship between per-capita supply and the proportion of the population diagnosed with schizophrenia or related disorders (*R*^2^ = 0.606). Meanwhile, the relationship between supply and the use of mental health and substance use disorder services in any location was *R*^2^ < 0.2. This result did not change when these services were disaggregated to hospitalizations related to mental illness or substance use disorders.

Locally weighted scatterplot smoothing curves (Figures A3 and A4 in the Supplemental Material) show the functional relationship between per capita supply and the use of psychiatric services. Except for psychiatric consultation, the curves were positive and nonlinear. We used simple linear regression models to estimate any psychiatric contact and ongoing psychiatric care as a function of the natural log of per capita supply. We used untransformed linear models for psychiatric consultation. Partial residual plots (Figures A5–A16 in the Supplemental Material) showed approximately linear functional relationships. Per capita supply was positively correlated with psychiatric contact in FY 2012/13 (*R*^2^ = 0.566) and FY 2021/22 (*R*^2^ = 0.290). Per capita supply was positively correlated with psychiatrist consultation in FY 2012/13 (*R*^2^ = 0.254) and weakly correlated with FY 2021/22 (*R*^2^ = 0.005). Finally, per capita supply positively correlated with ongoing psychiatric care in FY 2012/13 (*R*^2^ = 0.562) and FY 2021/22 (*R*^2^ = 0.411). In FY 2021/22, for the average region, each additional psychiatrist per 100,000 population was associated with 54 adults accessing psychiatric service per 100,000 population, 17 adults accessing psychiatric consultations per 100,000, and 43 adults accessing ongoing psychiatric care per 100,000. Note that in linear-log models, gains associated with additional per-capita supply are smaller for regions with higher supply. We performed the same analysis for the subpopulation of people diagnosed with schizophrenia and related disorders. In these analyses, per capita supply had a weaker linear correlation (*R*^2^ < 0.2) with all 3 measures of service use (see Table A3 in the Supplemental Material for full results).

**Figure 1.**
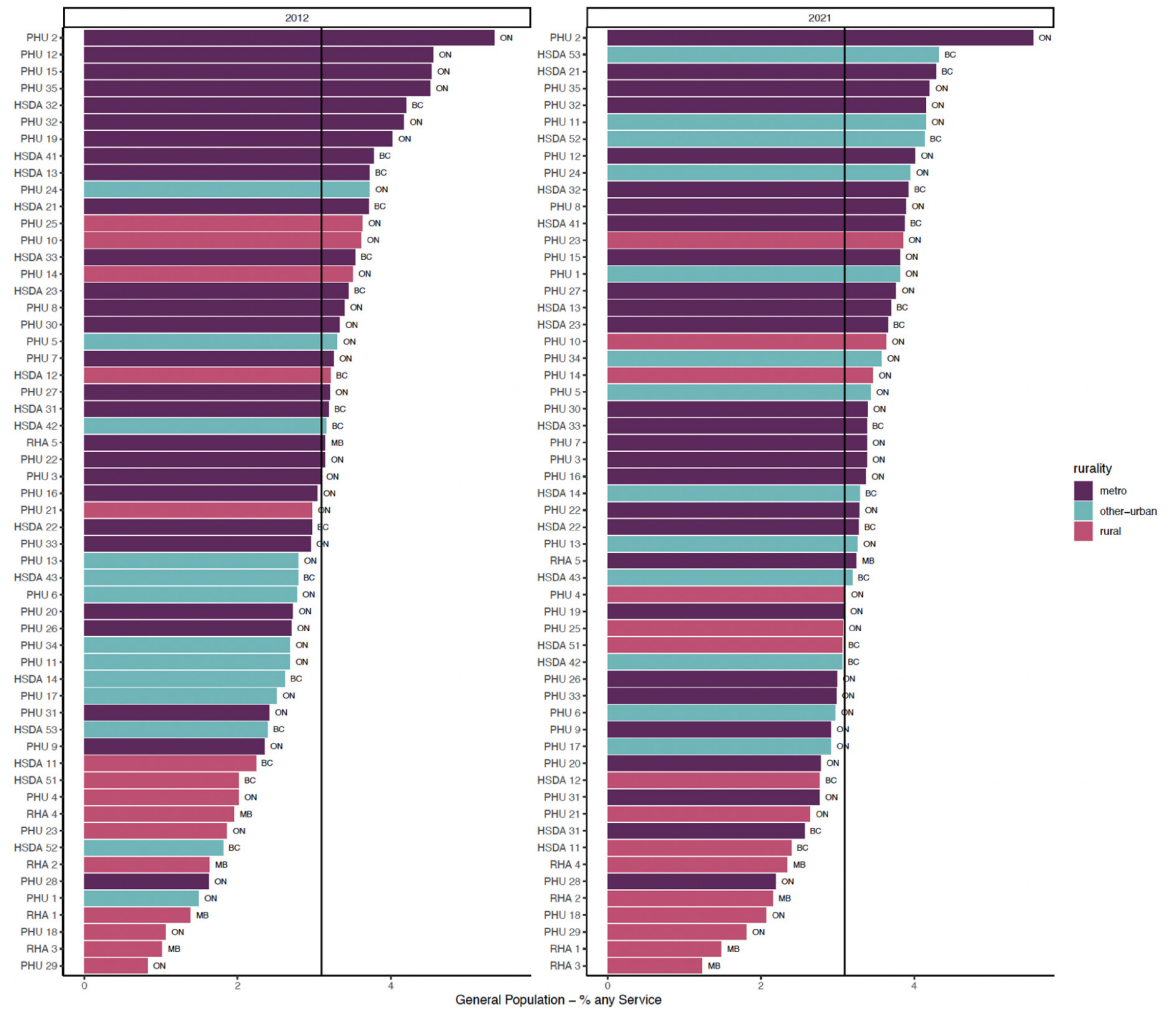
Proportion of general adult population with any psychiatrist contact.

**Figure 2.**
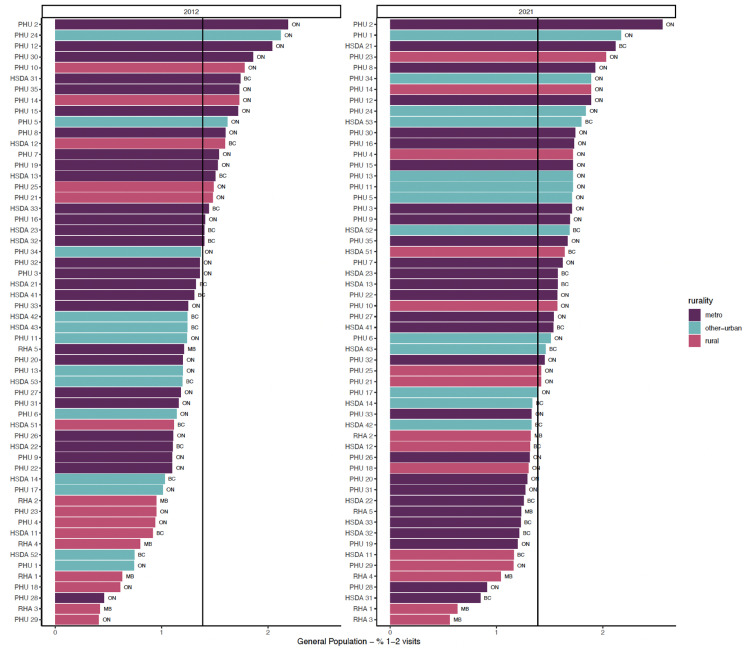
Proportion of general adult population with psychiatric consultation.

**Figure 3.**
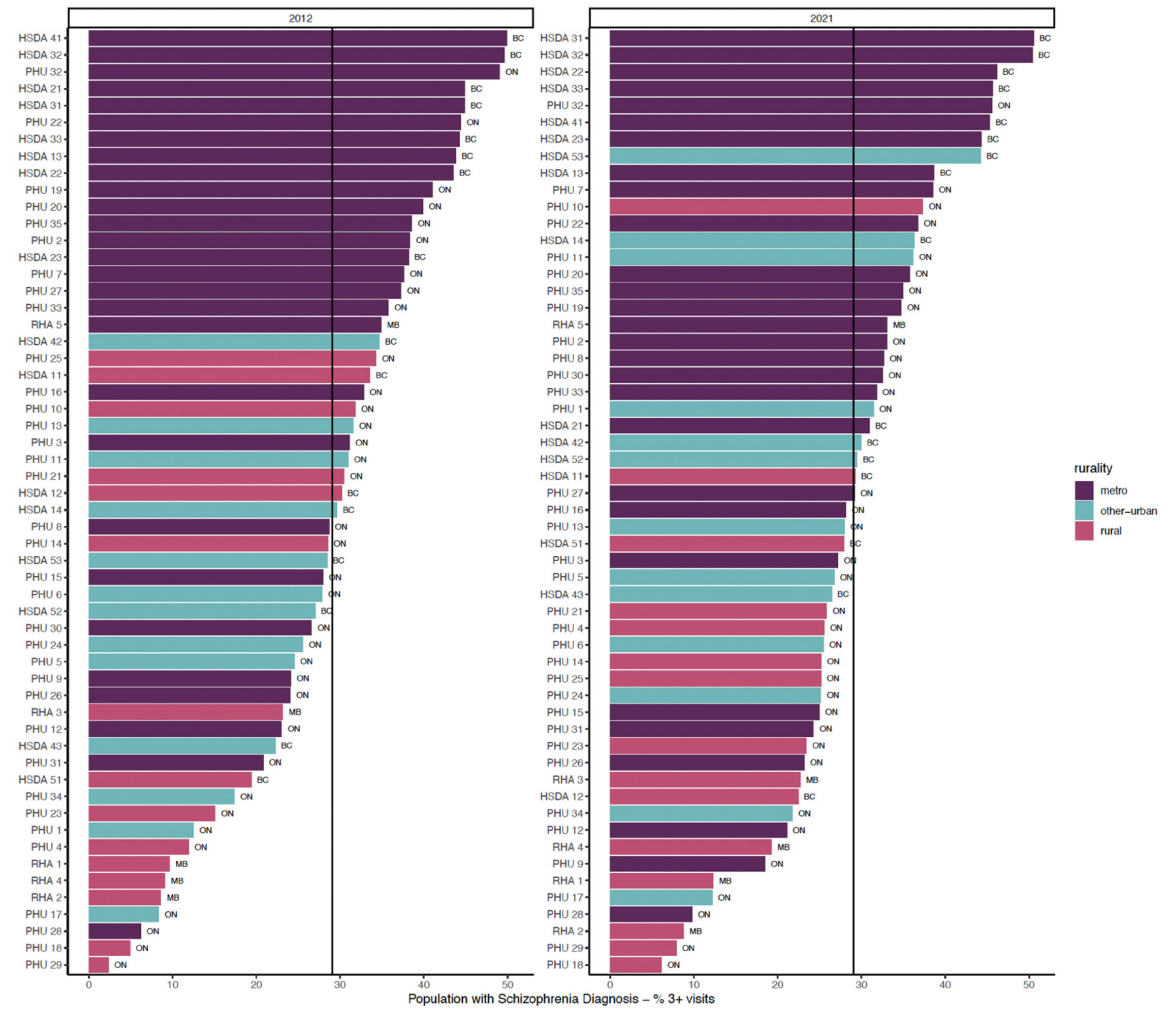
Proportion of general adult population with ongoing psychiatric care.

Figures 1–3 show regions ranked by 3 service variables: any psychiatric contact, psychiatric consultation, and ongoing psychiatric care. Table 2 displays means, standard deviations, and *R*^2^ values. In FY 2021/22, MB had the lowest rates overall: 20.88 per 1,000 for psychiatric contact, 9.56 per 1,000 for psychiatric consultation, and 8.42 per 1,000 for ongoing psychiatric care.

**Table 2.** Measures of use of Psychiatric Services by Province and by Rurality.

	FY 2012/13						FY 2021/22					
	Any contact per 1,000		Psychiatric consultation per 1,000		Ongoing care per 1,000		Any contact per 1,000		Psychiatric consultation per 1,000		Ongoing care per 1,000	
*Full population*	Mean	SD	Mean	SD	Mean	SD	Mean	SD	Mean	SD	Mean	SD
All regions in BC	30.470	6.911	12.700	2.547	14.240	4.699	34.294	5.899	14.426	2.982	15.837	3.818
All regions in Manitoba	18.220	8.132	8.020	3.016	7.460	5.583	20.880	7.906	9.560	3.456	8.420	5.562
All regions in Ontario	30.203	9.915	13.351	4.298	13.963	6.463	33.586	6.960	16.169	3.162	14.471	4.806
All metro regions	34.280	7.856	14.051	3.399	17.148	5.280	34.915	6.563	15.253	3.571	16.324	4.014
All other urban regions	26.688	5.785	12.228	3.581	11.285	2.897	35.417	4.838	16.588	2.455	15.452	3.902
All rural regions	21.928	9.713	10.547	4.603	8.467	5.376	26.038	7.759	13.451	4.096	9.603	4.299
*R*-squared: province*	0.133		0.140		0.096		0.241		0.278		0.158	
*R*-squared: rurality*	0.320		0.139		0.393		0.282		0.097		0.346	
*Schizophrenia diagnosis*	Mean	SD	Mean	SD	Mean	SD	Mean	SD	Mean	SD	Mean	SD
All regions in BC	580.505	69.772	153.803	44.199	365.307	96.846	594.487	70.717	149.329	28.328	373.994	93.384
All regions in Manitoba	369.000	136.128	120.060	61.533	170.680	116.768	396.420	111.934	129.700	65.940	191.940	95.012
All regions in Ontario	483.731	130.857	141.646	39.400	270.123	115.330	495.420	86.726	151.211	25.429	270.271	87.807
All metro regions	560.054	100.213	137.558	28.645	357.639	102.543	549.854	86.819	139.878	20.870	338.519	99.365
All other urban regions	470.690	110.884	156.039	48.754	246.804	77.301	522.441	87.639	167.295	25.015	287.216	77.430
All rural regions	417.547	145.230	142.575	58.598	195.352	113.663	443.063	106.112	149.251	44.319	212.717	88.441
*R*-squared: province^a^	0.241		0.045		0.213		0.327		0.038		0.289	
*R*-squared: rurality^a^	0.231		0.030		0.348		0.199		0.125		0.257	

^a^ Unweighted for population.

In FY 2021/22, there were varying rates of psychiatric contact based on the rurality levels. Rural and other urban areas had lower rates than metro regions. There was a positive relationship between rurality and any psychiatric contact (*R*^2^ = 0.282) and ongoing psychiatric care (*R*^2^ = 0.346). This relationship was weaker for psychiatric consultations (*R*^2^ = 0.097).

Regional rankings for the subpopulation of people diagnosed with schizophrenia or related disorders are provided in Figures A17–A19 in the Supplemental Material. In FY 2021/22, Manitobans with this diagnosis had lower rates of service use compared to those in BC and Ontario: 396.42 per 1,000 for psychiatric contact, 129.70 per 1,000 for psychiatric consultation, and 191.94 per 1,000 for ongoing psychiatric care.

## Discussion

This study found large variations in the number of psychiatrists (“supply”) and psychiatry services (“utilization”) within and across the 3 Canadian provinces. MB consistently had a lower per capita supply than ON and BC throughout the study period. Urban regions in all provinces tended to have a higher per capita supply. Specifically, Vancouver, Toronto, and Winnipeg were among the top 8 health regions in terms of the per capita number of psychiatrists in FY 2021/22. There was a strong correlation (*R*^2^ > 0.6) between regional supply and regional rates of diagnosis of schizophrenia and related disorders. Rates of schizophrenia and related disorders are consistently higher in more densely populated areas due to migration for services, higher environmental risk, and other factors.^19^ Otherwise, the number of psychiatrists was more weakly associated with crude measures of regional need for psychiatric services, such as hospitalization for mental disorders or substance use disorders. These findings align with those of previous research conducted in ON, which indicated that Toronto had the highest per capita number of psychiatrists, but there was no correlation between supply and crude measures of need.^13^

The uneven distribution of psychiatrists highlighted here is compounded by reports of a worsening shortage,^20^ especially as psychiatrists age.^16^ More psychiatrists will be required to meet the growing demand for their services, but recruitment could be challenged by provider burnout,^21,22^ and disparities in compensation, with psychiatrists being the lowest or second lowest paid medical specialty in Canada.^23^ Nonetheless, there are encouraging trends in psychiatrist recruitment, with more Canadian medical students choosing psychiatry as their area of specialization.^24^ The expansion of virtual care and increased funding for Internet-delivered psychotherapies^25^ also show promise in alleviating some of the maldistribution and access concerns.^26^ The transition to “telepsychiatry” occurred rapidly during the COVID-19 pandemic, providing access to psychiatric care during lockdown.^27^ These same strategies could offer an opportunity to enhance access to psychiatrists in rural communities. However, research is needed to understand how to incentivize psychiatrists to use virtual care to accept patients in lower supply regions of the country, and to understand the unintended consequences of increased use of virtual care, including potential access challenges for some populations.^26^ Other promising strategies to improve access to outpatient mental health services include increased use of computer-based psychotherapies,^28^ and psychotherapies delivered by counsellors, lay providers, and peer support workers.^29,30^

This study also found positive relationships between the number of psychiatrists and rates of service use, particularly ongoing psychiatric care. However, marginal increases in utilization diminish with a higher per capita supply. It is important to note that we did not capture information on full-time psychiatrist equivalents. Therefore, we were unable to distinguish between psychiatrists providing full-time clinical services and those who spent a significant proportion of their time on non-clinical work, such as research or administration. Future research that investigates psychiatrist practice patterns could differentiate between these psychiatrists and full-time practice. Nonetheless, previous research in Ontario suggests that in high-supply regions, psychiatrists are more likely to build practices with fewer patients and see them more often.^13,14^ If average practice sizes shrink as supply increases, this could explain the diminishing marginal increase in the number of people accessing psychiatric services. While ongoing psychiatric care may be beneficial for patients with complex needs who require ongoing monitoring and adjustment of medications, previous research also suggests that psychiatrists with smaller practices are less likely to see patients with more complex or urgent needs.^13–15^

In addition, this study found that more psychiatrists per capita did not correlate strongly with the rates of psychiatric consultations (measured as 1 or 2 contacts with the same psychiatrist). While we cannot interpret this result causally, it suggests that increasing supply alone may not lead to a higher utilization of consultation services. Improving access may also be realized through a shift in practice, where psychiatrists focus more of their attention on being accessible consultants to other healthcare providers.^31,32^ To support this shift, psychiatrists could practice in collaborative team-based mental health care models where they work closely with primary care providers to consult, educate, and in so doing, provide indirect care to individuals seen in primary care practices.^33^ They could still provide direct care for the most complex cases. These models have been demonstrated to be effective for patients with a variety of mental disorders,^34,35^ but have not been widely implemented in Canada.^36^ There are a number of barriers to this strategy, including traditional fee-for-service payment models and physician-centric public health insurance systems that can hinder interprofessional collaboration.^36,37^ The increased provision of consultation services also assumes that patients have access to a regular primary care provider who can implement consultation recommendations and provide ongoing mental healthcare. However, 1 in 7 Canadians does not have access to such a provider,^38^ and the disparities in access for some groups, including low-income populations, have been increasing over time.^39^ Those in need of specialized mental health services may rely on psychiatrists for ongoing care when primary care is not available, or when they do not have the means to pay for services outside the public insurance system. As a result of these barriers, increasing access to psychiatric services via practice changes will likely require more foundational changes in the organization of both the mental and primary healthcare sectors in Canada. It is also uncertain whether psychiatrists would accept a greater focus on consultation and how policy changes to promote this shift would affect psychiatrist recruitment and retention.

## Limitations and Strengths

This study has some limitations. First, we could only observe the use of psychiatric services received, as reflected in the administrative (billing) data; we cannot draw conclusions regarding the level of demand or unmet needs in the population, nor could we observe the use of mental health services delivered by other specialized mental healthcare providers. Second, administrative health data only capture patient contacts that were billed or shadow-billed via the provincial health insurance system. This may limit our ability to observe some services provided by salaried psychiatrists. Some salaried psychiatrists in all 3 provinces shadow bill; however, because their compensation is not linked to billing, some clinical activity may be underreported. Comparing the number of psychiatrists captured in our data with those registered with their respective provincial colleges, we found that approximately 10% of psychiatrists were unaccounted for in all 3 provincial datasets (Table A4). Since salaried psychiatrists in all 3 provinces would have similar incentives to report clinical activity in the billing data, we suspect that the data are similarly biased across provinces. Third, we were unable to describe the setting in which psychiatric care was used. For outpatient visits, contact with psychiatrists could have occurred in a range of settings, including hospitals and private offices. Furthermore, we did not consider the characteristics of the psychiatrist workforce. These supply-side factors could provide further insight into patterns of supply and use of psychiatric care. Fourth, we used crude measures of regional need for psychiatric services. Since these measures are based on the utilization of healthcare services (e.g., inpatient care), they cannot fully capture latent population needs. Fifth, we captured data on 3 provinces in Canada, which limits our ability to draw conclusions about the supply of psychiatric services across Canada.

This study has several strengths. We were able to access data from these 3 provinces and develop comparable indicators for the use of psychiatric services. We developed these indicators to differentiate between the core clinical activities that psychiatrists perform, such as psychiatric consultation and ongoing psychiatric care. Accessing administrative data allowed us to observe all psychiatric services billed under provincial health insurance plans over a 10-year period and track supply and use at the regional level. These strengths enabled us to highlight the large disparities in the distribution of psychiatric care within and across provinces.

## Conclusions

This study suggests that the current supply of psychiatrists is unevenly distributed within and across Canadian provinces. While more psychiatrists are needed to address maldistribution, diminishing returns to supply indicate that increasing the number of psychiatrists alone is unlikely to solve the problem of unmet need for mental healthcare. Strategies to improve access to care will need to directly target the uneven distribution of mental health care resources. This includes fundamental changes in how psychiatrists are supported and how they practice, such as improved infrastructure for virtual care, a shift to team-based mental healthcare models, and the use of evidence-based technology. Furthermore, healthcare systems need to be more involved in shaping psychiatric practice to focus on high-need populations through payment schemes, contracts, and other management levers. More research is needed to understand the factors that contribute to psychiatrists’ practice choices and the ways in which they can be better supported to increase access to care.

## Supplemental Material

sj-docx-1-cpa-10.1177_07067437251322404 - Supplemental material for Regional Variation in Supply and Use of Psychiatric Services in 3 Canadian Provinces: Variation régionale de l’offre de services psychiatriques et de leur utilisation dans trois provinces canadiennes Supplemental material, sj-docx-1-cpa-10.1177_07067437251322404 for Regional Variation in Supply and Use of Psychiatric Services in 3 Canadian Provinces: Variation régionale de l’offre de services psychiatriques et de leur utilisation dans trois provinces canadiennes by David Rudoler, Ridhwana Kaoser, M Ruth Lavergne, Sandra Peterson, James M. Bolton, Matt Dahl, François Gallant, Kimberley P Good, Myriam Juda, Alan Katz, Jason Morrison, Benoit H. Mulsant, Alison L Park, Philip G Tibbo, Juveria Zaheer and Paul Kurdyak in The Canadian Journal of Psychiatry
