# Supplementary material for: Regional Variation in Supply and Use of Psychiatric Services in 3 Canadian Provinces: Variation régionale de l’offre de services psychiatriques et de leur utilisation dans trois provinces canadiennes
Source: Can J Psychiatry. 2025 Mar 28;70(6):511–23. doi: 10.1177/07067437251322404 (PMC12185946; doi:10.1177/07067437251322404)
Supplement: sj-docx-1-cpa-10.1177_07067437251322404 - Supplemental material for Regional Variation in Supply and Use of Psychiatric Services in 3 Canadian Provinces: Variation régionale de l’offre de services psychiatriques et de leur utilisation dans trois provinces canadiennes [file sj-docx-1-cpa-10.1177_07067437251322404.docx]

## Supplemental Material

Table A1: Description of Datasets

| Dataset | BC | MB | ON |
| --- | --- | --- | --- |
| Physician registry | College of Physicians and Surgeons of British Columbia: Provides data on all registered physicians. | Provider Registry (Physician Master File): Captures provider details such as specialty, date of birth/age, training location, years of practice, accepted payment methods, workload, and practice groups/practice location. The file is updated quarterly. The Electronic User Site Location (EUSL) data is used to determine practice location. | Corporate Provider Database: Holds details of all physicians and some non-physician providers (like chiropractors, physiotherapists, and optometrists) funded by the Ministry of Health through OHIP or other funding. It covers demographics, eligibility, specialty, and practice locations. The ICES Physician Database: Holds annual data on all Ontario physicians. It includes details from the Ontario Health Insurance Plan (OHIP), Corporate Provider Database (CPDB), Ontario Physician Human Resource Data Centre (OPHRDC), and the OHIP physician billing database. |
| Physician billings | The Medical Services Plan (MSP): Collects data on medically necessary services offered by fee-for-service practitioners to individuals covered by BC’s universal insurance program. The data also contains encounter (shadow) claims if submitted by alternatively-paid practitioners. | Medical Claims/Medical Services Data: Health data from Manitoba Health includes claims for physician visits, tests in offices and hospitals, payments for on-call agreements (e.g. anesthesiologists) not linked to specific patients, and details on physician specialties. | Ontario Health Insurance Plan: ICES received claims data mostly from the Ontario Health Insurance Plan, covering all healthcare providers eligible to claim under OHIP, such as physicians, groups, laboratories, and out-of-province providers. |
| Hospital separations | The CIHI Discharge Abstract Database: Contains information on discharges, transfers, and deaths of inpatients and day surgery patients from acute care hospitals, including inpatient acute, chronic, and rehabilitation cases. | The CIHI Discharge Abstract Database: Contains information on discharges, transfers, and deaths of inpatients and day surgery patients from acute care hospitals, including inpatient acute, chronic, and rehabilitation cases. | The CIHI Discharge Abstract Database: Contains information on discharges, transfers, and deaths of inpatients and day surgery patients from acute care hospitals, including inpatient acute, chronic, and rehabilitation cases. Ontario Mental Health Reporting System (OMHRS): Provides information on all individuals who received adult mental health services in Ontario, which includes location of admission, discharge info, mental and physical health. |
| Emergency department visits | The CIHI National Ambulatory Care Reporting System: Covers all levels of ambulatory care in Canada, including emergency departments, day surgery, medical and surgical day clinics in hospitals, communities, and private clinics. In BC, NACRS data is used to capture Emergency Department visits from reporting facilities ( 30 EDs in 2021-22). The combination of 2 data sources, NACRS and Medical Services Plan (MSP), contains the majority of ED visits in BC. | The Emergency Department Information System: Stores data about patients in a hospital’s emergency department. It includes patient demographics, arrival details, initial assessments like chief complaint and vital signs, CTAS score, healthcare providers, treatment orders, consultations, discharge diagnosis, and status. | The CIHI National Ambulatory Care Reporting System: Covers all levels of ambulatory care in Canada, including emergency departments, day surgery, medical and surgical day clinics in hospitals, communities, and private clinics. |
| Consolidation file | The Central Demographic File (formerly known as Consolidation File): Population Data BC’s central demographics file for research requests. It contains basic demographics such as age and sex, geo-codes indicating location of residence, and registration data. | Manitoba Health Insurance Registry: The registry includes individual-level demographics, family composition information, residential postal codes, and data fields for registration, birth, entry into province, and migration in/out of province. Data contains all individuals who have been registered with Manitoba Health. | Registered Persons Database: Provides basic demographic information about anyone who has ever received an Ontario health card number. Data supplied by the Ministry of Health is enriched with information from other ICES datasets. |

Table A2: Description of Variables

| Variable | BC | MB | ON |
| --- | --- | --- | --- |
| Total population | Total number of general population aged 18-105 eligible for provincial health insurance within the fiscal year. | Total number of general population aged 18-105 eligible for provincial health insurance within the fiscal year. | Total number of general population aged 18-105 eligible for provincial health insurance within the fiscal year. |
| Number of psychiatrists | Physicians with who made one or more claims as a psychiatrist between 2012/2013 and 2021/2022 | Physicians with who made one or more claims as a psychiatrist between 2012/2013 and 2021/2022 | Physicians with who made one or more claims as a psychiatrist between 2012/2013 and 2021/2022 |
| Psychiatrists per 100 000 population. | Denominator for the calculation of the rate is the full population. | Denominator for the calculation of the rate is the full population. | Denominator for the calculation of the rate is the full population. |
| % MHSU users | Percent of adults 18-105 who had one of the following:   - Emergency department visits: DX10CODE1: ICD-10-CA F06 to F99); DX10CODE2 to DX10CODE3: X60-X84, Y10-Y19, Y28 - Hospital separations DX10CODE1: F06 to F99; DX10CODE10-25: X60-X84, Y10-Y19, Y28; DX10CODE1: 099.3, R45.8 - Physician billings: ICD9 chapter V (290 to 319), plus Self-Harm: E950-E959, plus anxiety/depression: 50B. Exclude dementia (ICD-9:290.x and 294.x) | Percent of adults 18-105 who had one of the following:   - Emergency department visits: DX10CODE1: ICD-10-CA: F06 to F99; DX10CODE2-10: X60-X84, Y10-Y19, Y28 - Hospital separations: DX10CODE1: F06 to F99; DX10CODE1-25: X60-X84, Y10-Y19, Y28; DX10CODE1: O99.3, R45.8. From Ontario Mental Health Reporting System: If var AXIS1_DSM4CODE_DISCH1 complete (i.e,. listed diagnosis from below present) use AXIS1_DSM4CODE_DISCH1: If not, use PROVDX1; exclude OMHRS admissions if AXIS1_DSM4CODE_DISCH1 in: (290.x OR 294.x): ICD-10-CA: F06 to F99; DSM-IV: Any (including missing diagnoses except for DSMIV 290.x. or 294.x in AXIS1_DSM4CODE_DISCH1) - Physician billings: ICD9 chapter V (290 to 319), plus Self-Harm: E950-E959, 959, 989, 977. Exclude dementia (ICD-9:290.x and 294.x) | Percent of adults 18-105 who had one of the following:   - Emergency department visits: From EDIS/ICD-10-CA:: DX10CODE1: ICD-10-CA F06 to F99; DX10CODE2 to DX10CODE3:: X60-X84, Y10-Y19, Y28 - Hospital separations: From DAD/ICD-10-CA codes: DX10CODE1: F06 to F99; DX10CODE1-25: X60-X84, Y10-Y19, Y28; DX10CODE1=O99.3, R45.8 - Physician billings: ICD-9 chapter V (291 to 319); plus Self-Harm: E950- E959. Exclude dementia (ICD-9 290 and 294) |
| Proportion lowest neighbourhood income quintile | Proportion of regional population in the lowest neighbourhood income quintile based on Census data. | Proportion of regional population in the lowest neighbourhood income quintile based on Census data. | Proportion of regional population in the lowest neighbourhood income quintile based on Census data. |
| Proportion age 18 - 44 | Proportion of the regional population aged between 18 and 44. | Proportion of the regional population aged between 18 and 44. | Proportion of the regional population aged between 18 and 44. |
| Substance use disorder service users per 1 000 | Rate of unique individuals aged 18-105 who received any treatment for substance use disorders. ICD9: 303, 304; DX10CODE1-NACRS: F10-19, F55, F63.0; DX10CODE1: F10-F19, F55, F63.0 | Rate of unique individuals aged 18-105 who received any treatment for substance use disorders. ICD9: 303, 304; DX10CODE1-NACRS: F10-19, F55, F63.0; DX10CODE1: F10-F19, F55, F63.0; OMHRS **see OMHRS tab**: 291.x (all 291 codes, excluding 291.82), 292.x (all 292 codes, excluding 292.85), 303.x (all 303 codes), 304.x (all 304 codes), 305.x (all 305 codes), 312.31. Provisional=4 | Rate of unique individuals aged 18-105 who received any treatment for substance use disorders. ICD9: 303, 304; DX10CODE1-EDIS: F10-19, F55, F63.0; DX10CODE1: F10-F19, F55, F63.0 |
| Schizophrenia diagnosis per 1 000 | Rate of unique individuals aged 18-105 with schizophrenia, schizoaffective disorder, or psychotic disorders NOS using the following algorithm: Flag individuals admitted to a hospital or received 3 or more claims (on different dates) in the physician claims data within a rolling 36-month period for schizophrenia, schizoaffective disorder or psychotic disorders NOS. Use the 5-year look-back to create this flag, including the data to the end of the current FY. E.g. for FY 2012-13, use 2007-08 to 2012-13 data (5-year lookback plus current year). Within that time frame, use rolling 36 month periods to check for 3+ outpatient visits (or 1 or more hospitalization)). Day surgeries NOT included.  Codes used in algorithm: ICD9: 295 and 298; DX10CODE1: F20, F25, and F29 | Rate of unique individuals aged 18-105 with schizophrenia, schizoaffective disorder, or psychotic disorders NOS using the following algorithm: Flag individuals admitted to a hospital or received 3 or more claims (on different dates) in the physician claims data within a rolling 36-month period for schizophrenia, schizoaffective disorder or psychotic disorders NOS. Use the 5-year look-back to create this flag, including the data to the end of the current FY. E.g. for FY 2012-13, use 2007-08 to 2012-13 data (5-year lookback plus current year). Within that time frame, use rolling 36 month periods to check for 3+ outpatient visits (or 1 or more hospitalization)). Day surgeries NOT included.  Codes used in algorithm: DX10CODE1: F20, F25, and F29; OMHRS: DSM-IV schizophrenia (295), schizoaffective  disorder (295), and psychotic disorders (298); ICD9: 295, 298 | Rate of unique individuals aged 18-105 with schizophrenia, schizoaffective disorder, or psychotic disorders NOS using the following algorithm: Flag individuals admitted to a hospital or received 3 or more claims (on different dates) in the physician claims data within a rolling 36-month period for schizophrenia, schizoaffective disorder or psychotic disorders NOS. Use the 5-year look-back to create this flag, including the data to the end of the current FY. E.g. for FY 2012-13, use 2007-08 to 2012-13 data (5-year lookback plus current year). Within that time frame, use rolling 36 month periods to check for 3+ outpatient visits (or 1 or more hospitalization)). Day surgeries that met the criteria were included.  Codes used in algorithm: ICD9: 295 and 298; DX10CODE1: F20, F25, and F29 |
| Mental health hospitalization per 10 000 | Regional rates of unique individuals admitted to a hospital psychiatric care for mental disorder. Codes: DX10CODE1= F06-F09, F20-F99 (excluding F55 and F63.0) OR DX10CODE2-DX10CODE25 = X60-X84, Y10-Y19, Y28 OR DX10CODE1=O99.3, R45.8 | Regional rates of unique individuals admitted to a hospital psychiatric care for mental disorder. Codes: DX10CODE1= F06-F09, F20-F99 (excluding F55 and F63.0) OR DX10CODE2-DX10CODE25 = X60-X84, Y10-Y19, Y28 OR DX10CODE1=O99.3, R45.8  OMHRS ALL Hospitalizations included - exclude if AXIS1_DSM4CODE_DISCH1 is any of the following (except as noted): 291.x (all 291 codes, keep 291.82), 292.x (all 292 codes, keep 292.85), 303.x (all 303 codes), 304.x (all 304 codes), 305.x (all 305 codes), 312.31. | Regional rates of unique individuals admitted to a hospital psychiatric care for mental disorder. Codes: DX10CODE1= F06-F09, F20-F99 (excluding F55 and F63.0) OR DX10CODE2-DX10CODE25 = X60-X84, Y10-Y19, Y28 OR DX10CODE1=O99.3, R45.8 |
| Substance use disorder hospitalization per 10 000 | Regional rates of unique individuals admitted to a hospital psychiatric care for substance use disorder. Codes: DX10CODE1: F10-F19, F55, F63.0 | Regional rates of unique individuals admitted to a hospital psychiatric care for substance use disorder. Codes: DX10CODE1: F10-F19, F55, F63.0  OMHRS: 291.x (all 291 codes, excluding 291.82), 292.x (all 292 codes, excluding 292.85), 303.x (all 303 codes), 304.x (all 304 codes), 305.x (all 305 codes), 312.31. Provisional=4 | Regional rates of unique individuals admitted to a hospital psychiatric care for substance use disorder. Codes: DX10CODE1: F10-F19, F55, F63.0 |
| Any psychiatrist contacts per 1 000 | Rate of unique patients seen by a psychiatrist (in any location, including office, emergency department, and hospital) to the adult population eligible for provincial health insurance within a region | Rate of unique patients seen by a psychiatrist (in any location, including office, emergency department, and hospital) to the adult population eligible for provincial health insurance within a region | Rate of unique patients seen by a psychiatrist (in any location, including office, emergency department, and hospital) to the adult population eligible for provincial health insurance within a region |
| Psychiatric consultations per 1 000 | Rate of unique patients with one or two outpatient visits with the same psychiatrist to the adult population within a region | Rate of unique patients with one or two outpatient visits with the same psychiatrist to the adult population within a region | Rate of unique patients with one or two outpatient visits with the same psychiatrist to the adult population within a region |
| Ongoing psychiatric care per 1 000 | Rate of unique patients with three or more outpatient visits annually with the same psychiatrist to the adult population within a region | Rate of unique patients with three or more outpatient visits annually with the same psychiatrist to the adult population within a region | Rate of unique patients with three or more outpatient visits annually with the same psychiatrist to the adult population within a region |
| Rurality | Grouped regions into “Metro”, “Other Urban” and “Rural” based on the plurality of regional residents living in the following Statistical Area Classification (SAC) categories: “Metro” (SAC type 1), “Other Urban” (SAC types 2 or 3), and “Rural” (SAC types 4 to 7) based on the predominant SAC types of regional residents | Grouped regions into “Metro”, “Other Urban” and “Rural” based on the plurality of regional residents living in the following Statistical Area Classification (SAC) categories: “Metro” (SAC type 1), “Other Urban” (SAC types 2 or 3), and “Rural” (SAC types 4 to 7) based on the predominant SAC types of regional residents | Grouped regions into “Metro”, “Other Urban” and “Rural” based on the plurality of regional residents living in the following Statistical Area Classification (SAC) categories: “Metro” (SAC type 1), “Other Urban” (SAC types 2 or 3), and “Rural” (SAC types 4 to 7) based on the predominant SAC types of regional residents |
|  |  |  |  |

*Table A3 - Results of linear model of relationship between psychiatrists per 100,000 and rates of the use of psychiatric care*

| Model | Average Marginal Effect | R-squared |
| --- | --- | --- |
| FY2012: Any psychiatrist contact per 1,000 | 0.985 | 0.566 |
| FY2012: Psychiatric consultation per 1,000 | 0.114 | 0.254 |
| FY2012: Ongoing psychiatric care per 1,000 | 0.667 | 0.562 |
| FY2021: Any psychiatrist contact per 1,000 | 0.540 | 0.290 |
| FY2021: Psychiatric consultation per 1,000 | 0.017 | 0.005 |
| FY2021: Ongoing psychiatric care per 1,000 | 0.428 | 0.411 |
| **Schizophrenia diagnosis** |  |  |
| FY2012: Any psychiatrist contact per 1,000 | 5.567 | 0.134 |
| FY2012: Psychiatric consultation per 1,000 | -0.492 | 0.056 |
| FY2012: Ongoing psychiatric care per 1,000 | 6.388 | 0.181 |
| FY2021: Any psychiatrist contact per 1,000 | 3.635 | 0.082 |
| FY2021: Psychiatric consultation per 1,000 | -0.677 | 0.138 |
| FY2021: Ongoing psychiatric care per 1,000 | 4.898 | 0.126 |

*Table A4 - Comparison of college registered physicians to those captured in administrative health data*

| Province | Registered with Provincial Regularly College (as of May 2024) | Captured in Administrative Data (FY 2021/22) |
| --- | --- | --- |
| British Columbia | 995 | 870 |
| Manitoba | 205 | 186 |
| Ontario | 2638 | 2339 |

*Figure A1 - Per Capita Supply**


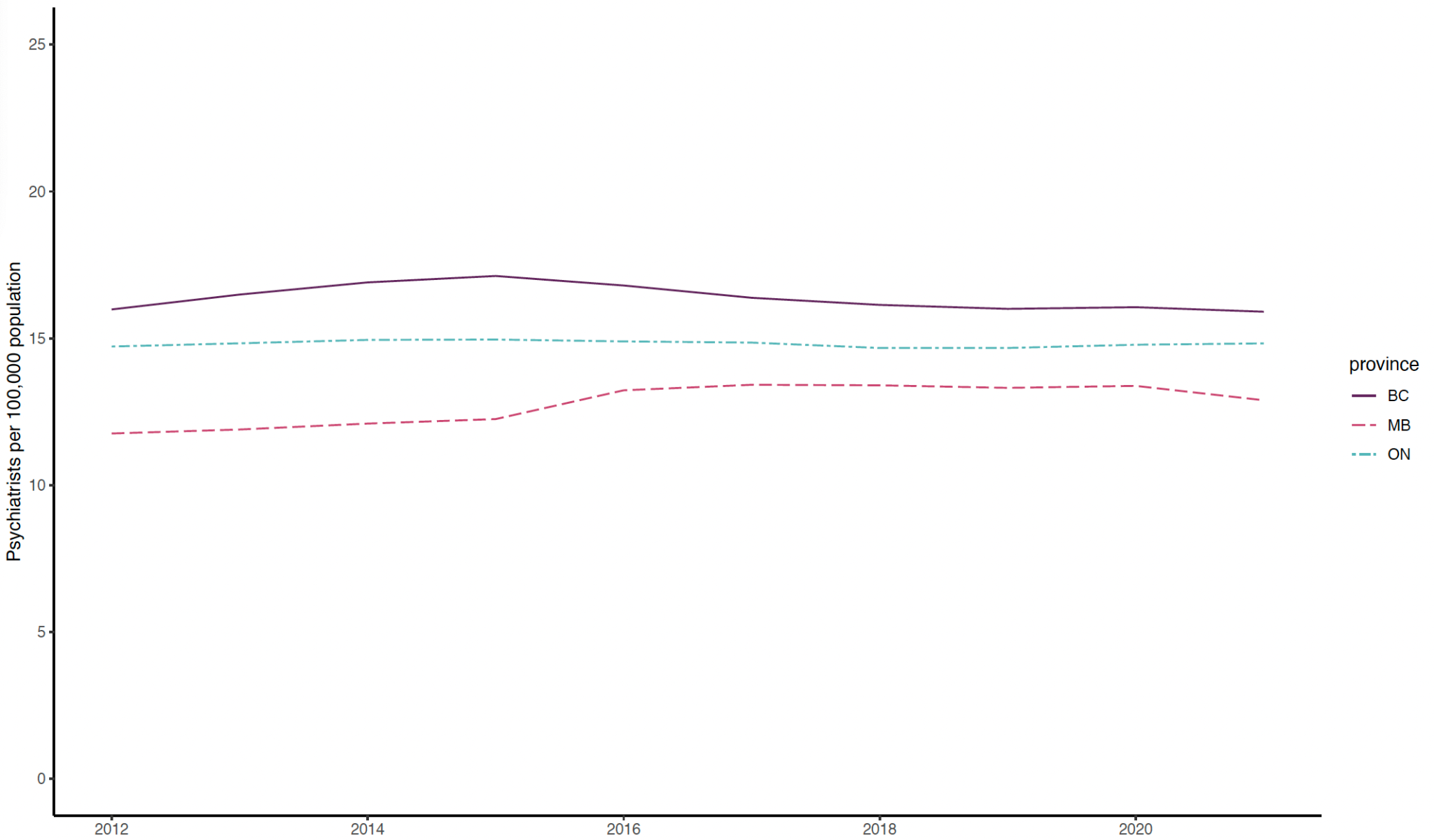


*Per-capita supply in the figure uses the full population denominator rather than the adult population (18–105 years).

*Figure A2 - Trends in use of psychiatric care (FY 2012/13 - FY 2021/22)*

*
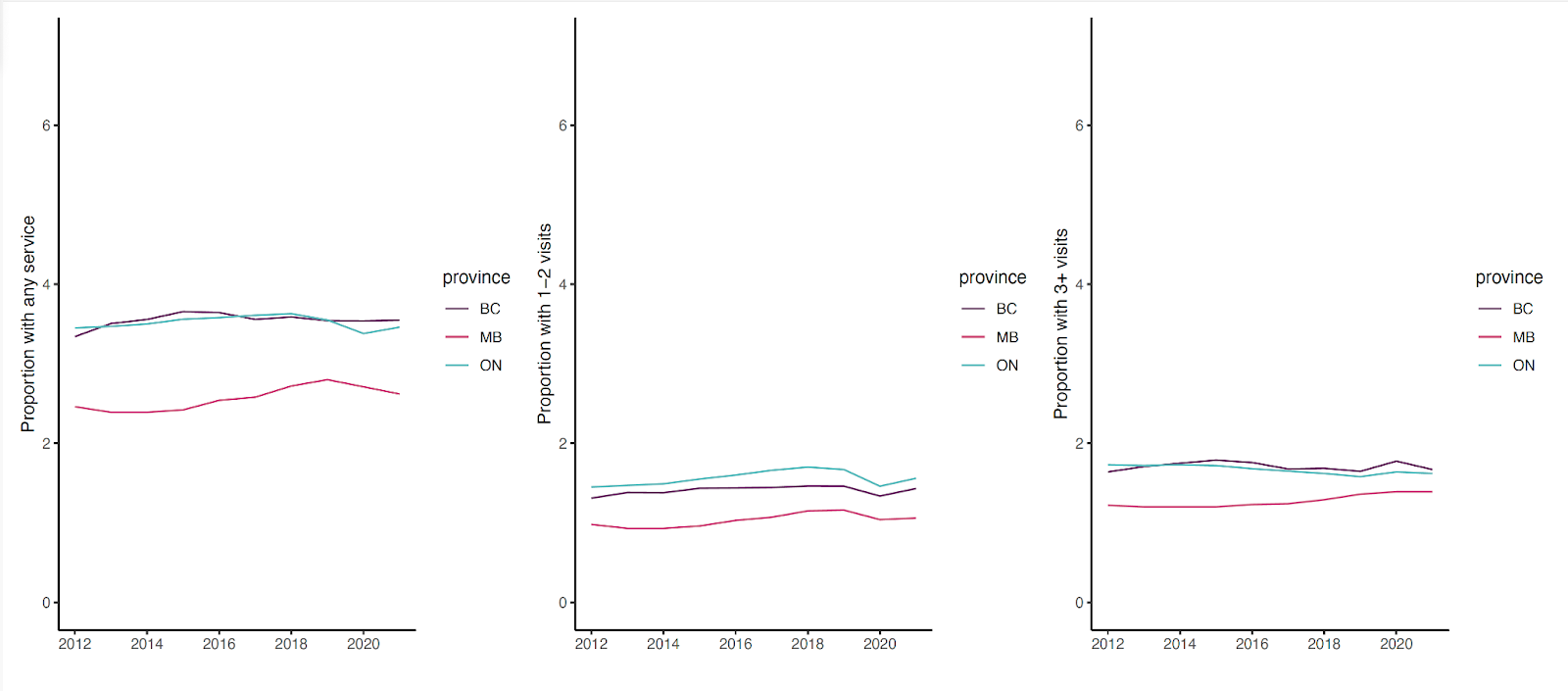
*

*Figure A3 - Locally weighted scatterplot smoothing curves of the relationship between psychiatrists per 100,000 and rates of the use of psychiatric care in FY 2012/13*


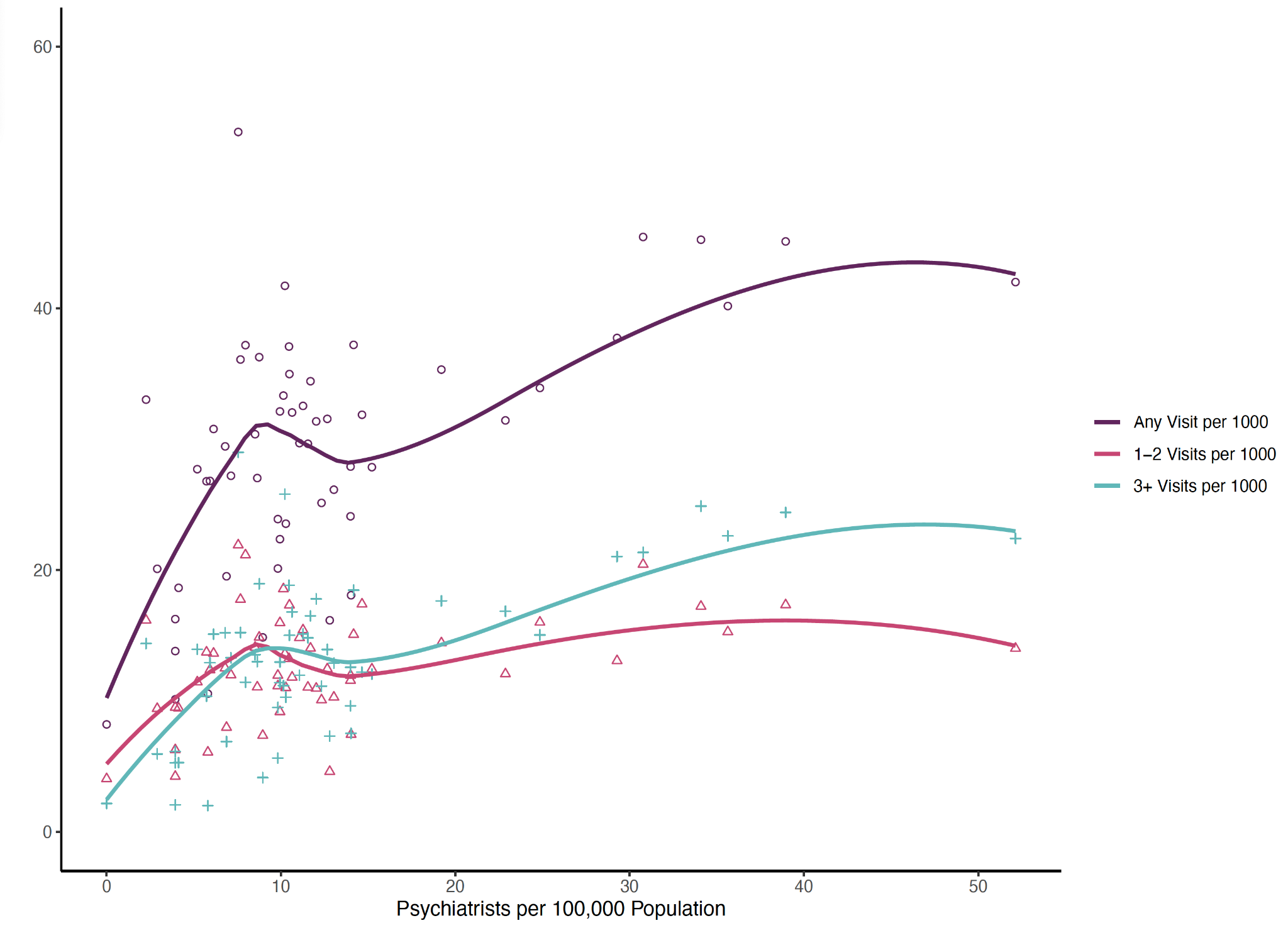


*Figure A4 - Locally weighted scatterplot smoothing curves of the relationship between psychiatrists per 100,000 and rates of the use of psychiatric care in FY 2021/22*

*
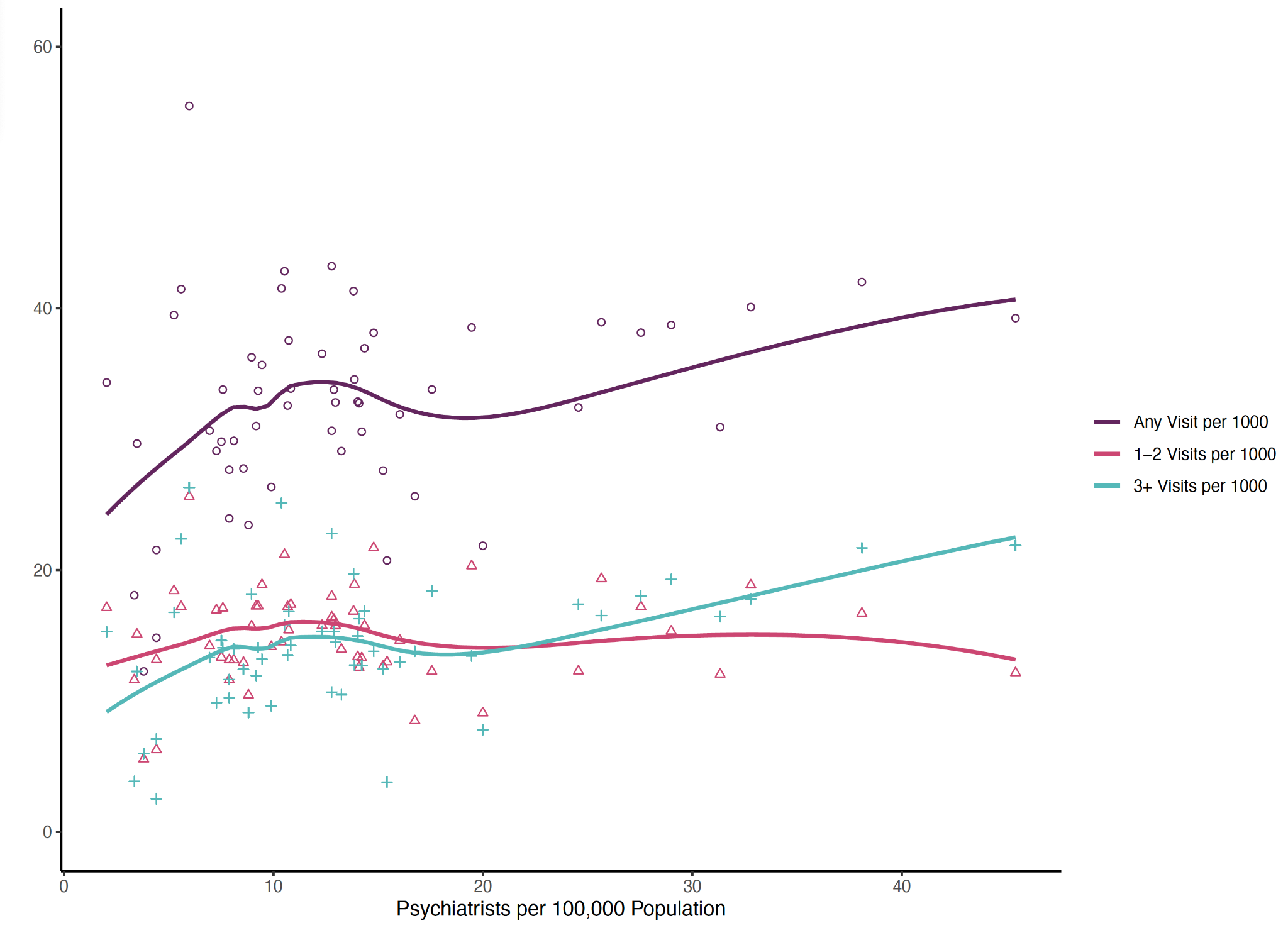
*

*Figures A5 – A7: Partial residual plots for the relationship between psychiatrists per 100,000 and rates of the use of psychiatric care in FY 2012/13*

*
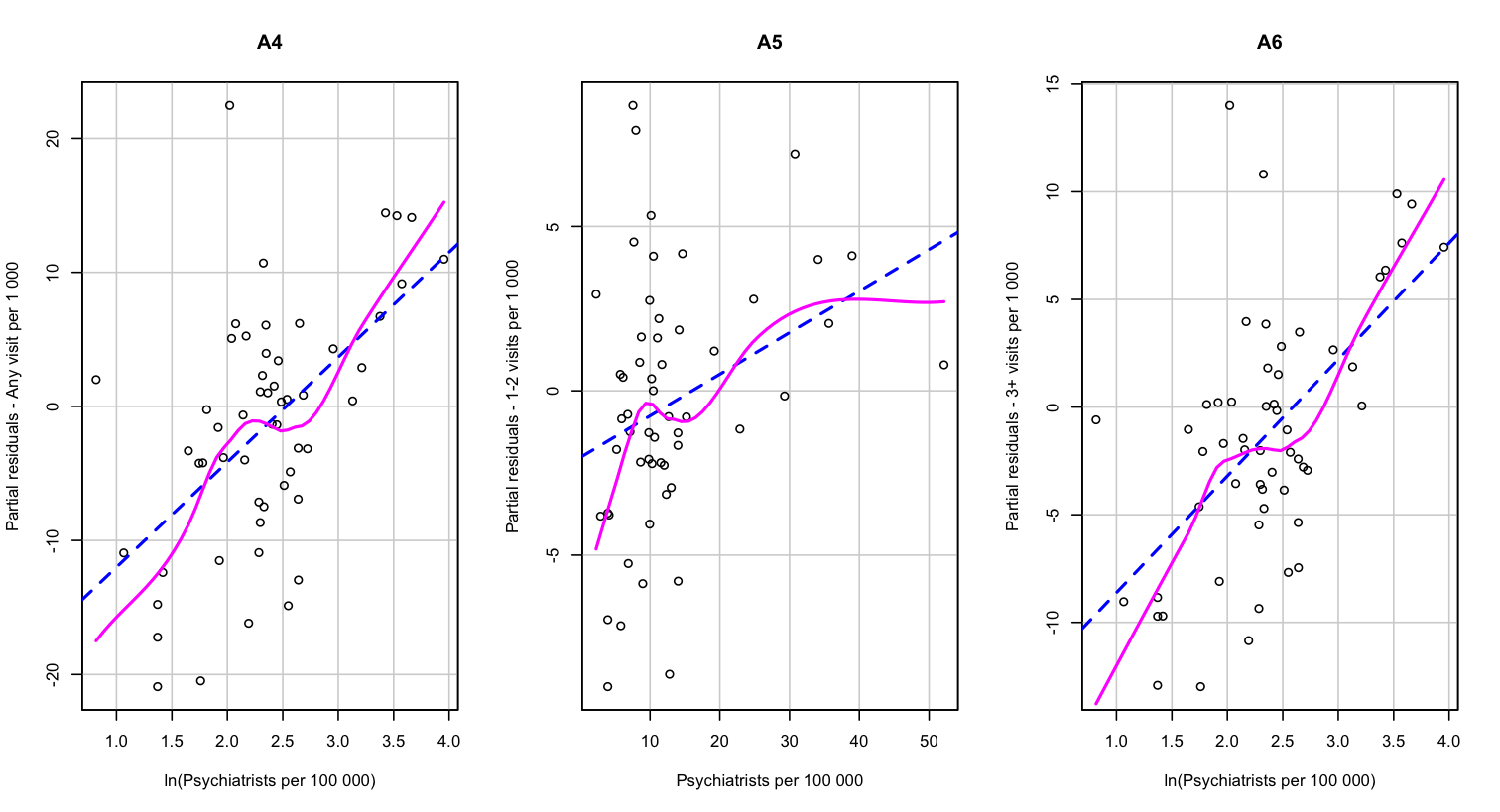
*

*Figures A8 – A10: Partial residual plots for the relationship between psychiatrists per 100,000 and rates of the use of psychiatric care in FY 2021/22*

*
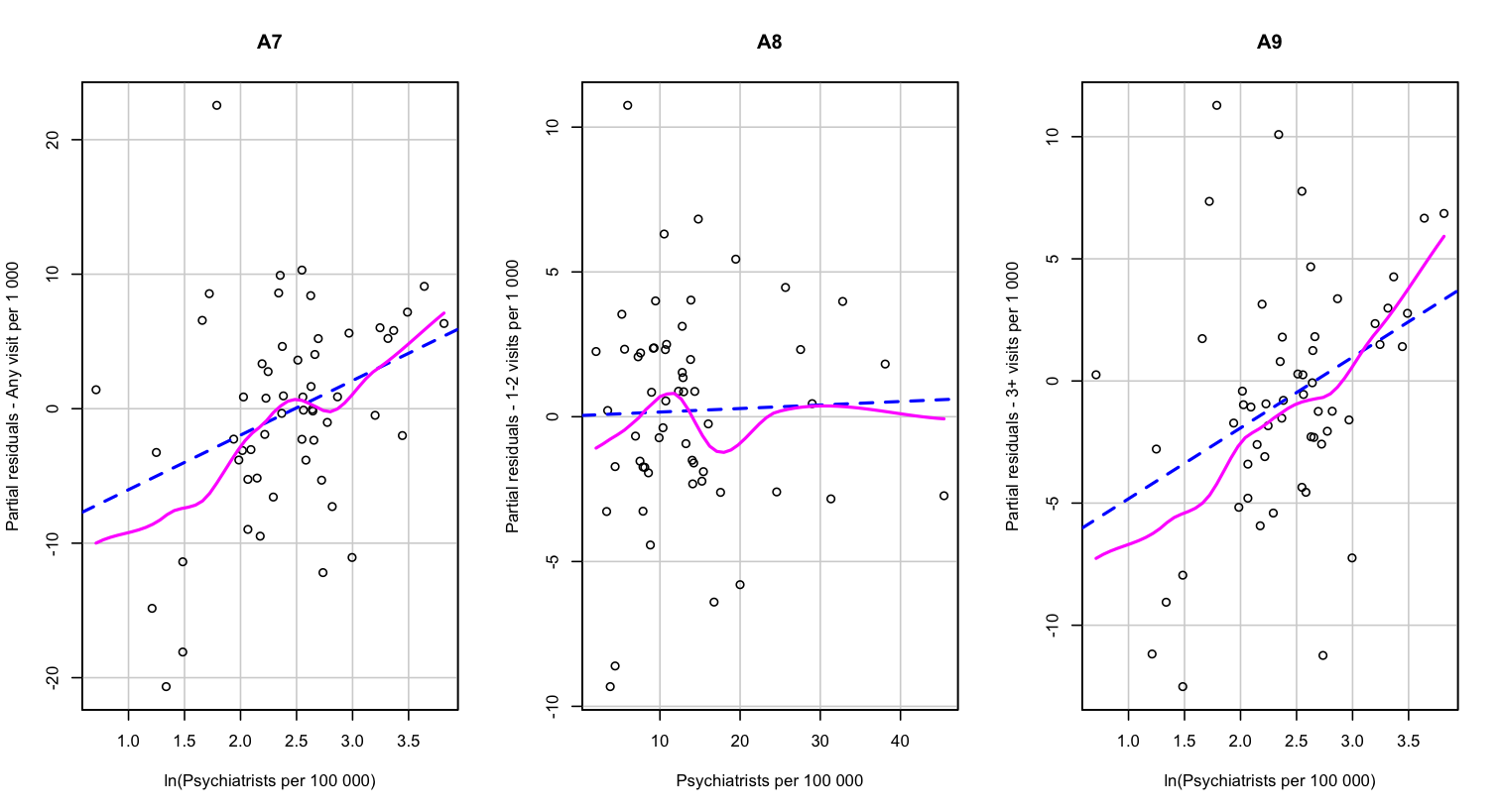
*

*Figures A11 - A13: Partial residual plots for the relationship between psychiatrists per 100,000 and rates of the use of psychiatric care in FY 2012/13 (sub-population of people with a schizophrenia diagnosis)*

*
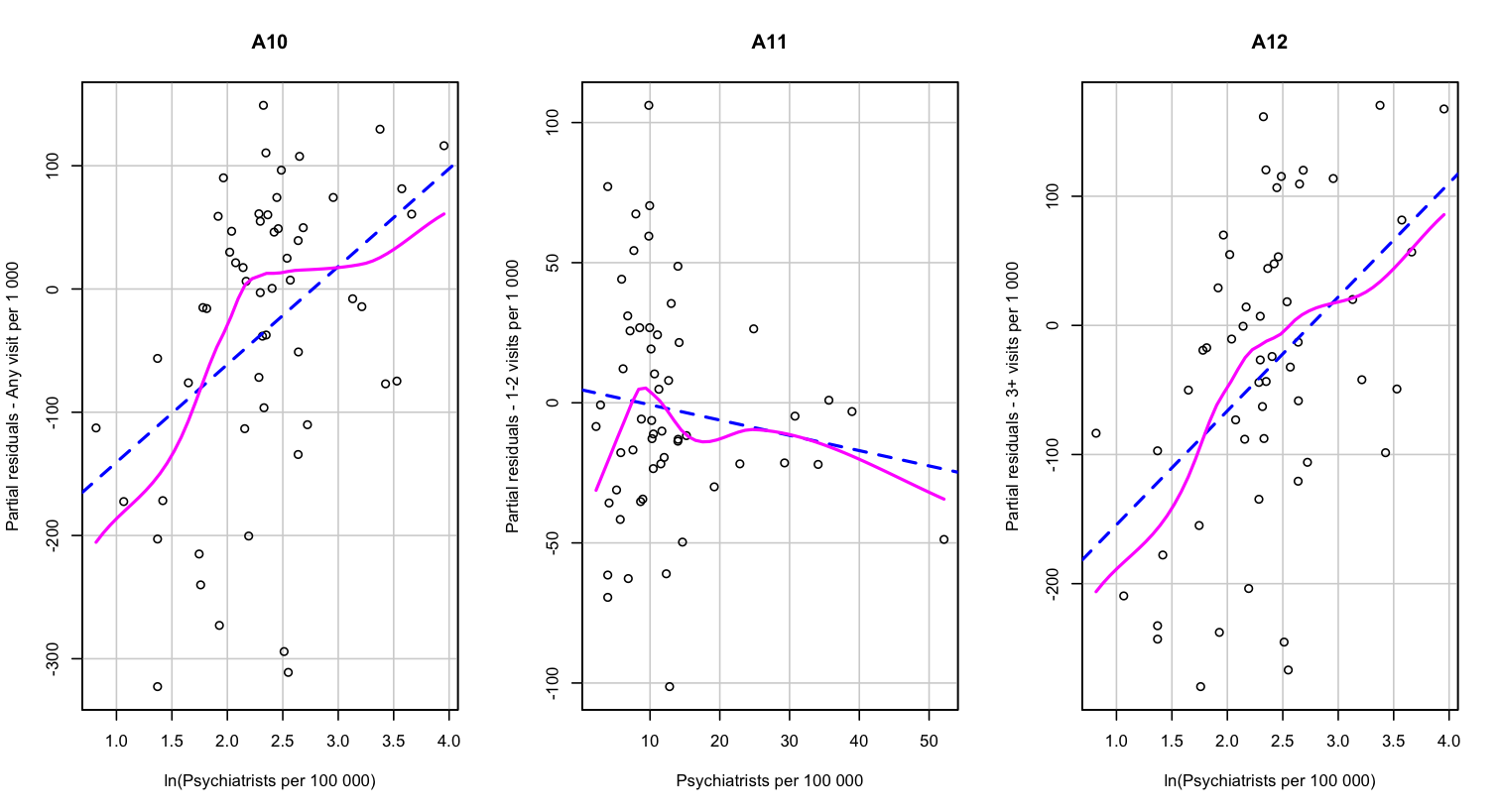
*

*Figures A14 - A16: Partial residual plots for the relationship between psychiatrists per 100,000 and rates of the use of psychiatric care in FY 2021/22 (sub-population of people with a schizophrenia diagnosis)*

*
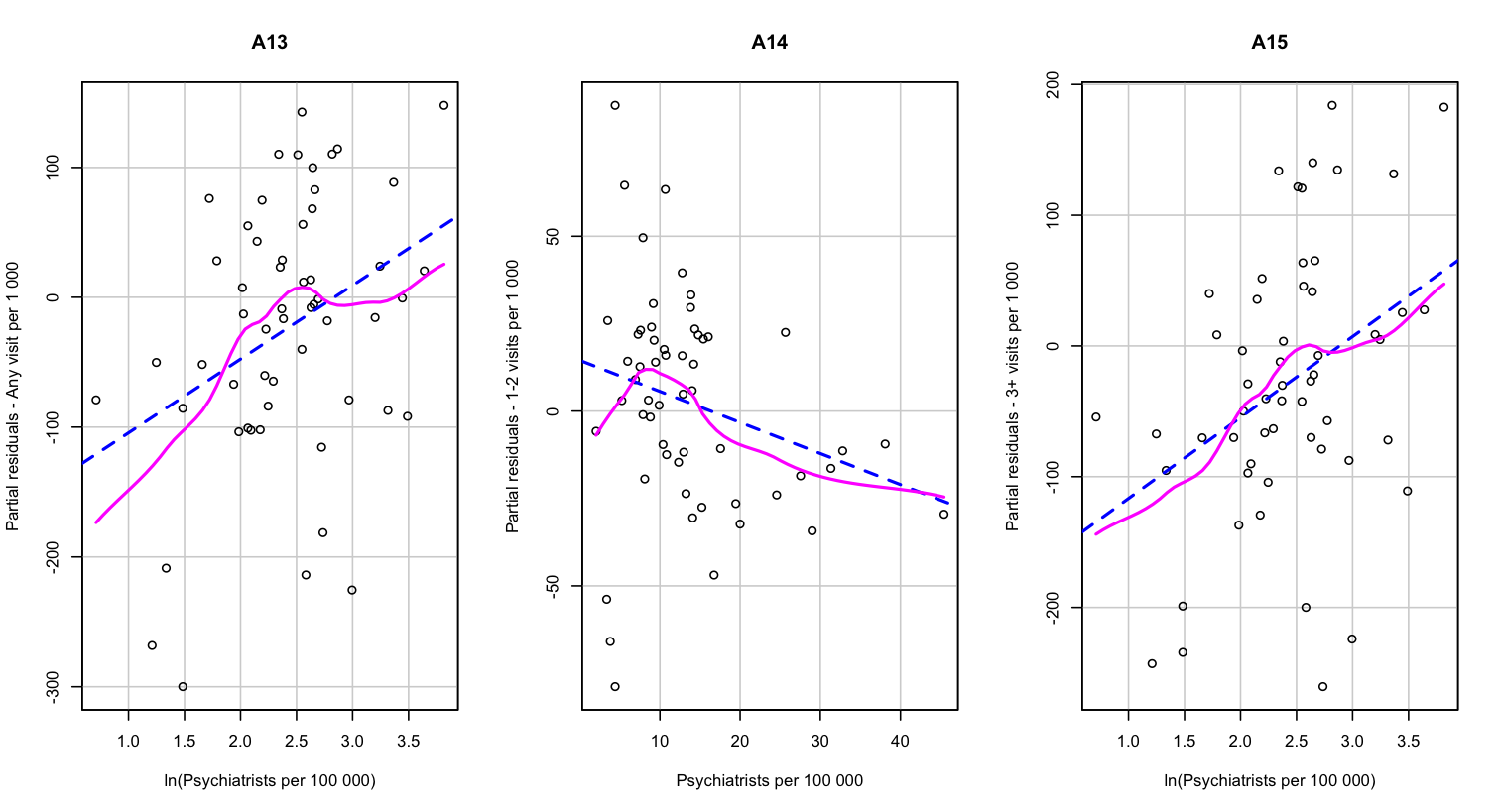
*

*Figure A17 - Proportion of population with schizophrenia diagnosis with any psychiatric service*


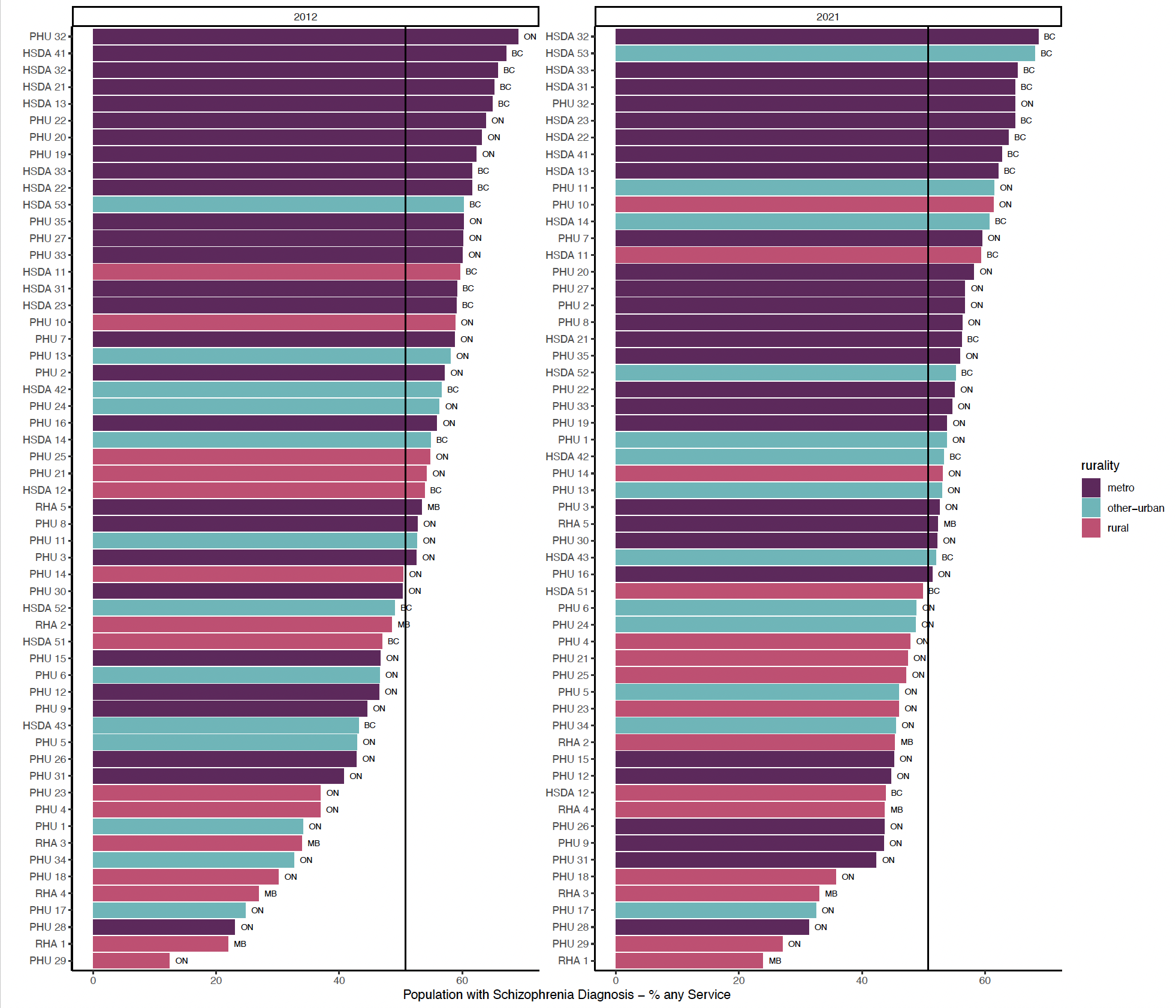


*Figure A18 - Proportion of population with schizophrenia diagnosis with psychiatric consultation*

*
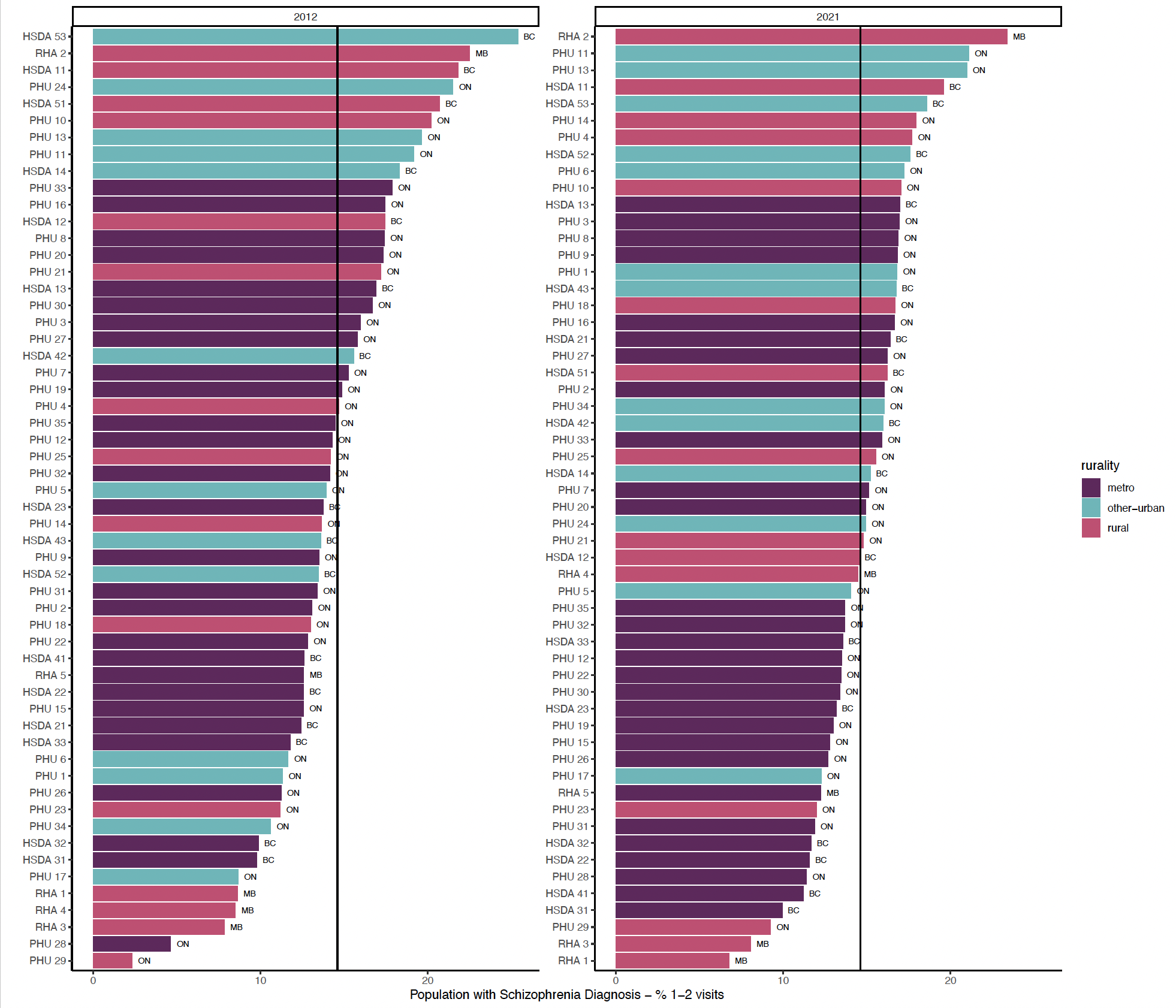
*

*Figure A19 - Proportion of population with schizophrenia diagnosis with ongoing psychiatric care*

*
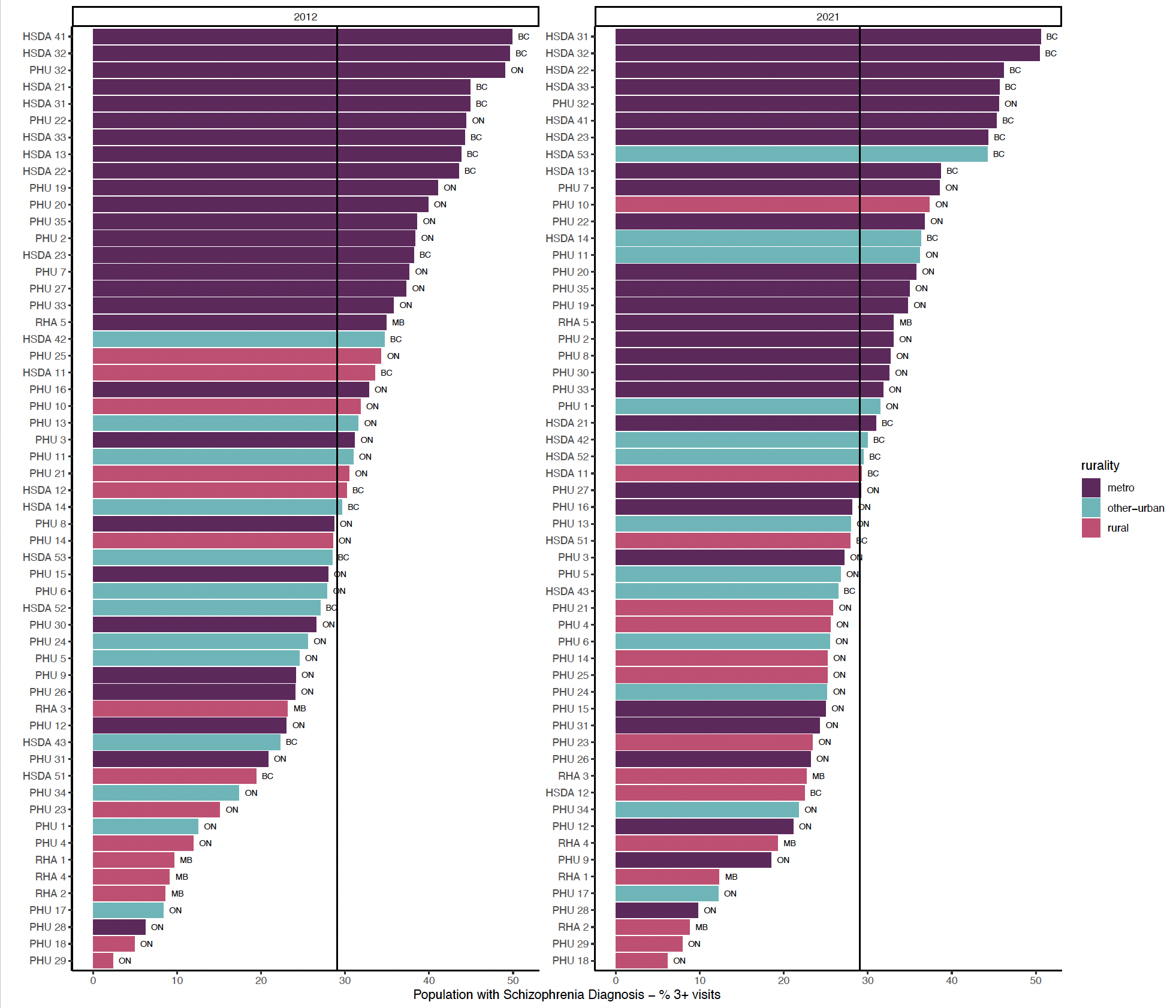
*
